# A study on the effect of different rifle calibres in euthanisation of grey seals (*Halichoerus grypus*) in seal traps in the Baltic Sea

**DOI:** 10.1186/1751-0147-55-79

**Published:** 2013-11-13

**Authors:** Torsten Mörner, Jonas Malmsten, Karin Bernodt, Sven-Gunnar Lunneryd

**Affiliations:** 1 Department of Pathology and Wildlife Diseases, National Veterinary Institute, 751 89 Uppsala, Sweden; 2 Department of Aquatic Resources, Swedish University of Agricultural Sciences, 453 21 Lysekil, Sweden

**Keywords:** Seals, *Halichoerus grypus*, Euthanasia, Rifles, Shotgun, Calibres

## Abstract

**Background:**

In recent years, the euthanasia of seals has been discussed internationally and concern has been raised regarding the use of rifles, the effect of different calibres, and which calibres are sufficient for humane euthanasia. This study therefore investigated the effect of different firearm calibres on euthanasia of grey seals (*Halichoerus grypus*) in traps, and provides information for the development and refinement of regulations for hunting seals in the wild.

**Findings:**

The effect of different calibres was studied in 19 seals shot in the head and neck at close range. All seals were necropsied and radiographed to characterize the injuries caused by the bullets. All tested calibres, 5.6 mm bullet diameter or larger, and .12 shotgun, were sufficiently effective to cause severe skull fractures, meningeal haemorrhages and instant death.

**Conclusions:**

Rifles with 5.6 mm bullet diameter or larger, and a .12 shotgun loaded with a slug fired at close range to the head and neck of grey seals all caused instant death and can therefore be recommended for hunting seals in the wild.

## Findings

Seal hunting and the euthanasia of seals have attracted much international interest in recent years, primarily regarding the harp seal (*Phoca groenlandica*) hunting in Atlantic Canada and its animal welfare aspects [1]. Seals killed on the ice in Canada are either shot at close range or clubbed with a so called hakapik, and the majority of the seals are killed in a humane manner [1].

A severe conflict between fisheries and the Baltic Sea population of Grey seal (*Halichoerus grypus*) has escalated during the last decades [2]. Hunting, one possible preventive management action, is now allowed in both Sweden and Finland using rifles of large calibres. Only calibres larger than 6.5 mm are permitted in Sweden.

**Figure 1:**
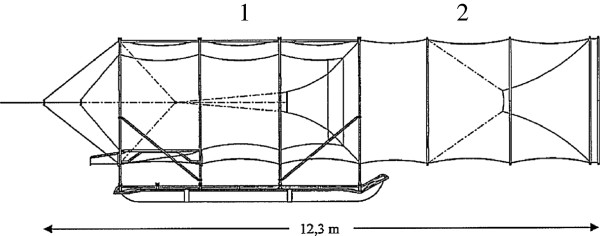
Side view of pontoon fish trap with entrance part (2) where the seals are caught, and the fish holding chamber (1) where the caught fish gather.

Net traps also are used to capture problem seals for euthanasia. These traps are additions to existing fishing gear where seals are caught (Figure 1) in a net cylinder chamber that floats on the water to ensure that seals are not drowned [3].

Previous studies has investigated the effect of and damage to the skull and brain using different calibres shooting at heads of dead harp seals [4], grey seals and harbour seals (*Phoca vitulina*) [5].

In this study 19 grey seals caught in net traps were euthanised with a shot to the centre of the head at close range (approximately 2–3 meters) by rifle or shotgun, following the recommendations for euthanasia of pinnipeds [6] (Table 1). We used the calibres .22 WM, with a bullet diameter of 5.6 mm; .222, with a bullet diameter of 5.7 mm; 7 × 57 with a bullet diameter of 7 mm; .30-06 and .308 with a bullet diameter of 7.62 mm; and calibre 12 shotgun slug, shown in Table 1. All rifle bullets used were hollow-point bullets. The study was performed to determine suitable and safe calibres for euthanising seals in a humane way that are also safe for hunters and not more powerful than needed.

**Table 1:** **Field data, calibre, number and location of shots, wind speed at time for euthanisation and pathological findings in euthanised grey seals ( *Halichoerus grypus *)**

**V-Nr**	**Date E.**	**Sex**	**Weight**	**Calibre**	**# shots**	**Pathological findings**	**Wind**
826/07	June 22	M	162 kg	.22 WM	2	1: Through mandible - NL	12 m/s
						2: Single fracture base skull - L	
918/07	July 6	M	123 kg	.22 WM	1	Fracture mandible, fracture base skull, intracranial haemorrhages - L	10 m/s
939/07	July 10	M	164 kg	.22 WM	2	1: Through nose and ethmoidal bone – NL	5 m/s
						2: Through right eye and front lobe of brain, multiple haemorrhages – L	
942/07	July 13	M	144 kg	.22 WM	1	Bullet through left eye and skull, fractures both sides skull - L	4 m/s
943/07	July 13	M	154 kg	30-06	1	Bullet through left eye and brain, multiple haemorrhages – L	4 m/s
944/07	July 14	M	172 kg	.308	1	Through front of head and the whole brain, out in neck – L	2 m/s
945/07	July 14	M	129 kg	.22 WM	1	Shot in neck, fracture allanto-occipital joint - L	4 m/s
946/07	July 15	M	120 kg	30-06	1	Through side of head, multiple fracture entire skull, brain haemorrhages – L	4 m/s
947/07	July 16	M	79 kg	7 X 57	1	Shot in neck, multiple fractures back skull, allanto-occipital joint – L	4 m/s
957/07	July 17	M	91 kg	.22 WM	1	Bullet through left eye, brain haemorraghes, fractures both sides skull - L	6 m/s
968/07	July 18	M	135 kg	.12 slug	1	Bullet through eye, brain haemorrhages, multiple fracture entire skull - L	8 m/s
969/07	July 19	M	130 kg	.22 WM	5	1: Bullet through nose and ethmoidal bone – NL	10 m/s
						2: Bullet in beyond eye, fracture both sides, brain multiple haemorrhages – L 3,4,5: Shots missed the animal	
970/07	July 23	M	122 kg	.12 slug	1	Bullet through eye, brain haemorrhages, fractures both sides skull - L	4 m/s
971/07	July 23	F	122 kg	.22 WM	1	Bullet in behind eye, brain haemorrhages, fractures both sides skull - L	2 m/s
985/07	July 25	M	138 kg	.22 WM	2	1: Bullet in behind eye, brain haemorrhages, fractures both sides skull – L	0 m/s
						2: Missed the head	
986/07	July 28	M	139 kg	.222	1	Shot in the neck, brain haemorrhages, multiple fractures back part of skull - L	6 m/s
987/07	July 28	M	153 kg	.12 slug	1	Bullet through eye, brain haemorrhages, fractures of the whole skull - L	4 m/s
988/07	July 29	M	118 kg	.12 slug	1	Bullet in below eye, brain haemorrhages, fractures of the whole skull - L	4 m/s
1001/07	August 2	M	119 kg	.22 WM	1	Bullet in below eye, brain haemorrhages, fractures and out in atlas region - L	10 m/s

L = lethal shot, NL = non lethal shot.

All 19 seals were examined at the NVI for lesions. The skull and neck were examined for injuries including fractures, haemorrhages and damage caused to the brain and meninges. The heads from 12 seals were radiographed to determine the exact path of the bullets through the head.

The lateral side of the skull of grey seals was 2–5 mm thick and the overlying muscles where bullets had entered were between 5 and 20 mm thick.

Field data, calibre, number and location of all shots, wind speed at time of euthanasia and pathological findings in euthanised seals are shown in Table 1. Information is also given as to whether or not the shot was lethal. All 19 seals had skull or neck fractures, and adjacent brain damage and/or severe meningeal or intracranial haemorrhages caused by the bullet.

**Figure 2:**
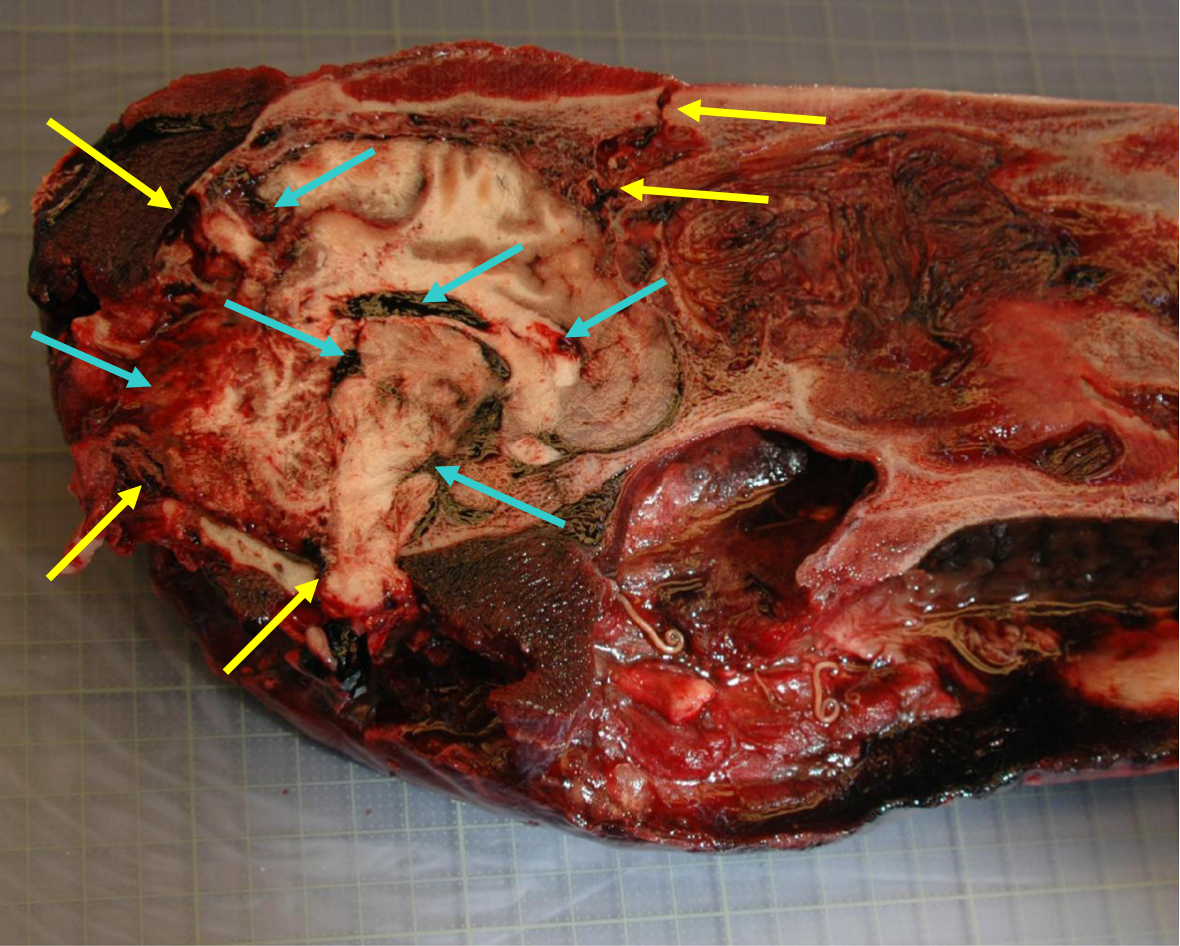
**Seal skull with injuries caused by .22 WM.** Note the fractures in the skull bone and the neck region (yellow arrows) and haemorrhages in the brain and meninges (Blue arrows).

In all five seals shot with calibres .222 or larger and in four seals shot with .12 shotgun slugs, multiple fractures in the skull, meningeal haemorrhages, and severe damage to the brain tissue were observed. In all 10 seals shot with .22 WM, single fractures were observed in the skull or the neck region causing lesions to the brain and spinal cord of the same character as in seals shot with larger calibres (Figure 2).

In four seals more than one shot was fired, all with .22 WM. Two of these seals were hit with both shots. One was wounded in the mandible, and the other in the nose. In the two other seals, only one shot hit the animal.

The wind speed was 10 m/sec or more in two instances when more than one shot was used, indicating that this should be taken into consideration when euthanising seals in traps.

All seals sustained injuries significant to cause immediate unconsciousness followed by rapid death. This study demonstrates that a rifle with calibre .222 or larger, and a 12 shotgun loaded with a slug all had the capacity to immediately euthanise the seals. It also clearly demonstrates that rifles with 5.6 mm bullet diameter or larger, fired at close range to the head and base of skull/upper neck of gray seals all caused instant death and can therefore be regarded as ballistically sufficient for euthanasia of adult seals in the wild.

## Abbreviations

WM: Winchester magnum; EPA: Swedish Environmental Protection Agency; NVI: National Veterinary Institute; SLU: Swedish University of Agricultural Sciences.

## Competing interests

The authors declare that they have no competing interests.

## Authors’ contributions

TM participated in the design of the study, in the post mortem examination of all animals and drafted the manuscript. KB and JM participated in the post mortem examination of all animals and helped draft the manuscript. SGL was responsible for all fieldwork and the euthanasia of animals and helped to draft the manuscript. All authors read and approved the final version of the manuscript.
